# Construction and verification of a risk prediction model for diabetic retinopathy patients complicated with deep capillary plexus diabetic macular ischaemia

**DOI:** 10.3389/fendo.2026.1681383

**Published:** 2026-01-29

**Authors:** Bo Li, Yanjun Du, Jianhong Li, Liying Yan, Chen Xie, Juan Xie, Yunchun Zou

**Affiliations:** 1 Nanchong Hospital of Capital Medical University Affiliated Beijing Anzhen Hospital · Nanchong Central Hospital Ophthalmology, Nanchong, China; 2 Suining Third People's Hospital Cardiovascular Internal Medicine, Suining, China; 3 Suining central hospital ophthalmology, Suining, China; 4 Ophthalmology and optometry medical college of North Sichuan medical college, Nanchong, China

**Keywords:** deep capillary plexus, diabetes, diabetic macular ischaemia, diabetic retinopathy, optical coherence tomography angiography, risk prediction model

## Abstract

**Background:**

To explore the risk factors for deep capillary plexus diabetic macular ischaemia (DCP-DMI) in patients with diabetic retinopathy and to establish and validate a risk prediction model.

**Methods:**

Diabetic retinopathy patients who visited the ophthalmology department of Suining Central Hospital were selected as the study subjects and divided into a DCP-DMI group and a non-DCP-DMI group based on the presence or absence of DCP-DMI. Independent risk factors for DCP-DMI in DR patients were screened through univariate analysis and binary logistic regression analysis. Draw a nomogram to show the risk prediction model, and internal validation was performed using the Bootstrap resampling method, while external validation was conducted using the time period validation method. The clinical application value of the model was assessed using decision curve analysis (DCA).

**Results:**

Binary logistic regression analysis revealed that glucose excursion, duration, hypertension, proliferative diabetic retinopathy (PDR), deep capillary plexus vascular density (DCP-VD), deep capillary plexus geometric perfusion deficit (DCP-GPD), and choroidal vascular density (CVD) are independent risk factors for DCP-DMI in DR patients. The area under the ROC curve of the risk prediction model was 0.918 (95% CI: 0.893–0.943), and the Hosmer–Lemeshow test showed P = 0.573. The area under the ROC curve for internal validation was 0.915 (95% CI: 0.911–0.918), and the Hosmer–Lemeshow test showed P = 0.262. The area under the ROC curve for external validation was 0.793 (95% CI: 0.741–0.846), and the Hosmer–Lemeshow test showed P = 0.246. The DCA decision curves for the model group and validation group indicated that the red line representing the column diagram model was above the two special reference lines, and there was a certain net benefit at different thresholds.

**Conclusions:**

Glucose excursion, duration, hypertension, PDR, DCP-VD, DCP-GPD, and CVD are closely related to the occurrence of DCP-DMI in DR patients. The column diagram model established on this basis has certain clinical reference significance.

## 1 Introduction

According to International Diabetes Federation data, the number of patients with diabetes worldwide reached 537 million in 2021, and it is expected to grow to 700 million by 2045. Diabetic retinopathy (DR) is a degenerative neurovascular disease of the retina secondary to diabetes (www.idf.org). Among patients with diabetes, the incidence of DR is as high as 35%, making it the leading cause of blindness in this population (1). The early causes of vision loss in patients with DR mainly include diabetic macular oedema (DME) and diabetic macular ischaemia (DMI), with DMI being more harmful, as its occurrence often indicates irreversible vision decline (2–4). DMI is a severe complication of DR and is characterized by microvascular occlusion and insufficient blood perfusion in the macular region (5). Since the macula is a key area for central vision, ischaemia directly damages retinal photoreceptor cells and bipolar cells, leading to irreversible central vision decline, visual distortion, and reduced contrast sensitivity (6). In addition, DMI can also affect the efficacy of anti-vascular endothelial growth factor (anti-VEGF) treatment for DME (7).

Fundus fluorescein angiography (FFA) is the most commonly used method to assess diabetic macular ischaemia (DMI) by observing the morphology of the foveal avascular zone (FAZ) (diameter ≥ 0.6 mm or area ≥ 0.5 mm²), the interruption of the capillary network in the macula, or the destruction of the arcades to define the presence of macular ischaemia (8). However, FFA has some obvious drawbacks as an examination method. First, as an invasive procedure, it carries the risk of drug allergies and is contraindicated in patients with liver and kidney dysfunction, pregnancy, and asthma, which limits its range of use (9). At the same time, FFA is a two-dimensional imaging technique that can only observe the vascular morphology of the superficial capillary plexus (SCP) in the macula and cannot assess the condition of the deep capillary plexus (DCP) and choroid, nor can it provide specific quantitative values (10). The DCP serves as the main oxygen source for photoreceptors in the macular area and has no effective compensatory pathways during ischaemia. The degree of damage to neurons and photoreceptors during diabetic retinopathy (DR) is more severe than that during SCP ischaemia (11). Additionally, DCP vessels have a smaller diameter, lower pericyte density, and a relatively singular blood supply, making ischaemia occur earlier in DR patients compared to SCP patients (12). Given the vascular structural network of the DCP, DCP-DMI holds an important research position in DR, but few studies have targeted DCP-DMI. Optical coherence tomography angiography (OCTA) is a fast, non-invasive, and dye-free technique that can achieve layered recognition of the retinal and choroidal vascular systems by obtaining three-dimensional data and can provide specific measurement values. It is now widely used in the assessment of retinal blood flow information (13). For DR, OCTA can measure the FAZ area, vascular density (VD), non-perfusion area, and vascular morphology indices of the SCP and DCP, as well as the thickness and VD of the choroid, allowing for the quantification of the severity and progression rate of DR for easier assessment and detection (14).

Although DMI poses a serious threat to the vision of DR patients, there is currently no clear treatment method (15). The only feasible treatment plan seems to be actively exploring and managing the associated risks. Currently, these measures mainly include the control of blood sugar and blood pressure (16). There are relatively few risk prediction studies on the occurrence of DMI in DR patients, and given that DCP-DMI in the macular region is more harmful than SCP-DMI, our study utilizes OCTA to collect relevant blood flow data while combining systemic factors to establish a risk predictive model for the occurrence of DCP-DMI in patients with DR, with the goal of providing a clinical reference for the prevention and tracking of MI in DR patients.

## 2 Methods

### 2.1 Patients and study design

This study was approved by the hospital ethics committee (approval number: KYLLKS20250136). A retrospective analysis was conducted. Patients with diabetic retinopathy (DR) who visited the ophthalmology centre of Suining Central Hospital from September 2020 to September 2023 were included in the model group. Patients with DR who visited the same centre from October 2023 to July 2025 were included in the validation group. OCTA images of a 3×3 mm^2^ area of the macular region were analysed for relevant parameters. Choroidal vascular density (CVD) refers to the vascular density of the choriocapillaris layer, which is the main source of blood supply to the foveal avascular zone (FAZ). The inclusion criteria were as follows: diagnosis of type 2 diabetes mellitus complicated by DR; diagnostic data for DMI obtained from OCTA according to FFA diagnostic criteria (There was capillary loss in the FAZ and/or there was non-perfusion area in macular area); OCTA signal quality ≥7; OCTA images exhibiting diabetic macular oedema (DME) and localized haemorrhage, causing retinal layering interference, manual adjustment of the OCTA layering interface to ensure accurate boundary positioning of the superficial capillary plexus (SCP) and deep capillary plexus (DCP). The exclusion criteria were as follows: incomplete clinical data; history of glaucoma or other retinal diseases; systemic diseases such as uraemia, end-stage renal disease, or malignant tumours; and history of intraocular surgery. Patients were divided into a DCP-DMI group and a non-DCP-DMI group based on the presence or absence of DCP-DMI. Independent predictive factors were determined by univariate analysis and multivariate logistic regression analysis, followed by establishment of a nomogram. The model was validated internally and externally using the Bootstrap method and time-period validation, respectively, and its clinical value was assessed through decision curve analysis (DCA).

### 2.2 Sample size calculation

Based on the relevant literature, clinical experience, and principles of avoiding feature redundancy in clinical prediction model parameters, 15 candidate predictive parameters were selected: glucose excursion, age, smoking (active smoking only), hypertension, proliferative DR (PDR), retinal laser (pan-retinal photocoagulation excluding macular grid laser), anti-VEGF therapy, DME, high myopia, SCP-DMI, DCP vascular density (DCP-VD), DCP geometric perfusion deficit (DCP-GPD), DCP adjusted blood flow index (DCP-AFI), and CVD. The sample size was calculated using the 10-events-per-predictor (EPP) rule. Given the 15 candidate parameters, 150 outcome events (15×10) were required. Considering a 30% incidence of DCP-DMI among DR patients, the total sample size required was 500 cases (150 ÷ 30%). The external validation sample size was calculated similarly, yielding 334 cases.

### 2.3 Statistical analysis

Statistical analysis was performed using SPSS 26.0 software. Normally distributed quantitative data are expressed as the mean ± standard deviation (x ± s), and inter-group comparisons were conducted using the independent samples t-test. Non-normally distributed quantitative data are expressed as the median and interquartile range M (P25, P75), with comparisons conducted using the Mann-Whitney U test. Count data are presented as frequency (n) and percentage (%) and were compared using the chi-square test. Binary logistic regression analysis was conducted to identify independent predictive factors. Differences with P < 0.05 were considered statistically significant. Model construction, testing, and evaluation of clinical application value were performed using RStudio software.

## 3 Results

### 3.1 Univariate analysis of DR patients with DCP-DMI

To ensure robustness during model construction, only samples with complete data were included (500 cases: 150 DCP-DMI and 350 non-DCP-DMI). During model validation using the time-period validation method, 13 cases had missing glucose excursion data among the 334 cases, since the missing rate was less than 5%, no imputation or special treatment was applied. A total of 15 factors were compared between the DCP-DMI and non-DCP-DMI groups. These factors included glucose excursion, age, duration of diabetes, smoking, hypertension, PDR, retinal laser treatment, diabetic macular edema (DME), anti-vascular endothelial growth factor (anti-VEGF) therapy, high myopia, SCP microinfarction (SCP-DMI), DCP vessel density (DCP-VD), DCP global perfusion deficit (DCP-GPD), DCP area of flow impairment (DCP-AFI), and cardiovascular disease (CVD). Univariate analysis revealed that glucose excursion, duration of diabetes, hypertension, PDR, DME, DCP-VD, DCP-GPD, and CVD were significantly associated (P < 0.05) (**Table 1**).

**Table 1:** Univariate analysis of DR patients with DCP-DMI.

Parameters	DCP-DMI group	non-DCP-DMI group	Statistical value (X^2^value/Z value)	P
Glucose excursion [n (%)]			18.891^1)^	<0.001
No	56(0.37)	205(0.59)		
Yes	94(0.63)	145(0.41)		
Age [Years,M(P25,P75)]	58(51-64)	56(50-64)	-1.175^2)^	0.240
Duration [Years,M(P25,P75)]	8(5-10)	3(1-6.25)	-9.638^2)^	<0.001
Smoking [n (%)]			2.562^1)^	0.109
No	68(0.45)	186(0.53)		
Yes	82(0.55)	164(0.47)		
Hypertension [n(%)]			14.986^1)^	<0.001
No	64(0.43)	215(0.61)		
Yes	86(0.57)	135(0.39)		
PDR [n(%)]			52.389^1)^	<0.001
No	57(0.38)	253(0.72)		
Yes	93(0.62)	97(0.28)		
Retinal laser [Times,M(P25,P75)]	2(0-4)	2(0-4)	-1.273^2)^	0.203
DME [n(%)]			5.702^1)^	0.017
No	74(0.49)	213(0.61)		
Yes	76(0.51)	137(0.39)		
anti-VEGF [Times,M(P25,P75)]	2(1-3)	1(0-2)	-1.685^2)^	0.092
High myopia [n(%)]			0.872^1)^	0.350
No	126(0.84)	305(0.87)		
Yes	24(0.16)	45(0.13)		
SCP-DMI [M(P25,P75)]			0.607^1)^	0.436
No	87(0.58)	216(0.62)		
Yes	63(0.42)	134(0.38)		
DCP-VD [M(P25,P75)]	0.36(0.33-0.39)	0.45(0.39-0.49)	-11.242^2)^	<0.001
DCP-GPD [M(P25,P75)]	1.40(1.29-1.56)	1.39(1.24-1.53)	-2.558^2)^	0.011
DCP-AFI [M(P25,P75)]	0.64(0.54-0.76)	0.67(0.56-0.79)	-1.680^2)^	0.093
CVD [M(P25,P75)]	0.56(0.51-0.59)	0.57(0.52-0.61)	-2.273^2)^	0.023

1)Pearson X^2^ test, 2)Mann-Whitney U test.

### 3.2 Multivariate analysis of DR patients with DCP-DMI

Based on the univariate analysis, the above eight significant factors were included in the binary logistic regression analysis. To avoid multicollinearity among independent variables, multicollinearity diagnostics were performed before logistic regression. The results showed tolerance values greater than 0.1 and variance inflation factors (VIF) less than 10, indicating no multicollinearity among variables (**Table 2**). The variables were then analysed using forward stepwise binary logistic regression. The analysis identified seven indicators as independent risk factors for DCP-DMI in DR patients: glucose excursion (OR = 3.0207, 95% CI: 1.5488–6.0976), duration of diabetes (OR = 2.7740, 95% CI: 1.9640–4.0400), hypertension (OR = 3.2514, 95% CI: 1.6848–6.4531), PDR (OR = 3.1658, 95% CI: 1.6202–6.3444), DCP-VD (OR = 0.1679, 95% CI: 0.1010–0.2622), DCP-GPD (OR = 1.4159, 95% CI: 0.9960–2.0555), and CVD (OR = 0.7761, 95% CI: 0.5538–1.0772) (**Table 3**).

**Table 2:** Collinearity analysis of risk factors for DCP-DMI in patients with DR.

Parameters	Collinearity statistics	
	Tolerance	Variance inflation factor
Glucose excursion	0.973	1.027
Duration	0.937	1.068
Hypertension	0.982	1.018
PDR	0.925	1.081
DME	0.985	1.015
DCP-VD	0.907	1.102
DCP-GPD	0.986	1.015
CVD	0.994	1.006

**Table 3:** Binary logistic regression of risk factors for DCP-DMI in patients with DR.

Parameters	β	SE	Wald	P	OR	95%CI	95%CI
Glucose excursion	0.9571	0.2862	3.34	0.0008	3.0207	1.5488	6.0976
Duration	1.0902	0.1548	7.04	<0.0001	2.7740	1.9640	4.0400
Hypertension	0.9681	0.2875	3.37	0.0008	3.2514	1.6848	6.4531
PDR	1.2990	0.2855	4.55	<0.0001	3.1658	1.6202	6.3444
DME	0.3099	0.2851	1.09	0.2770	1.3463	0.6921	2.6235
DCP-VD	-1.6291	0.1959	-8.27	<0.0001	0.1679	0.1010	0.2622
DCP-GPD	0.3371	0.1563	2.16	0.0310	1.4159	0.9960	2.0555
CVD	-0.3518	0.1403	-2.51	0.0122	0.7761	0.5538	1.0772

### 3.3 Construction of a risk nomogram model for DCP-DMI occurrence in DR patients

**Figure 1:**
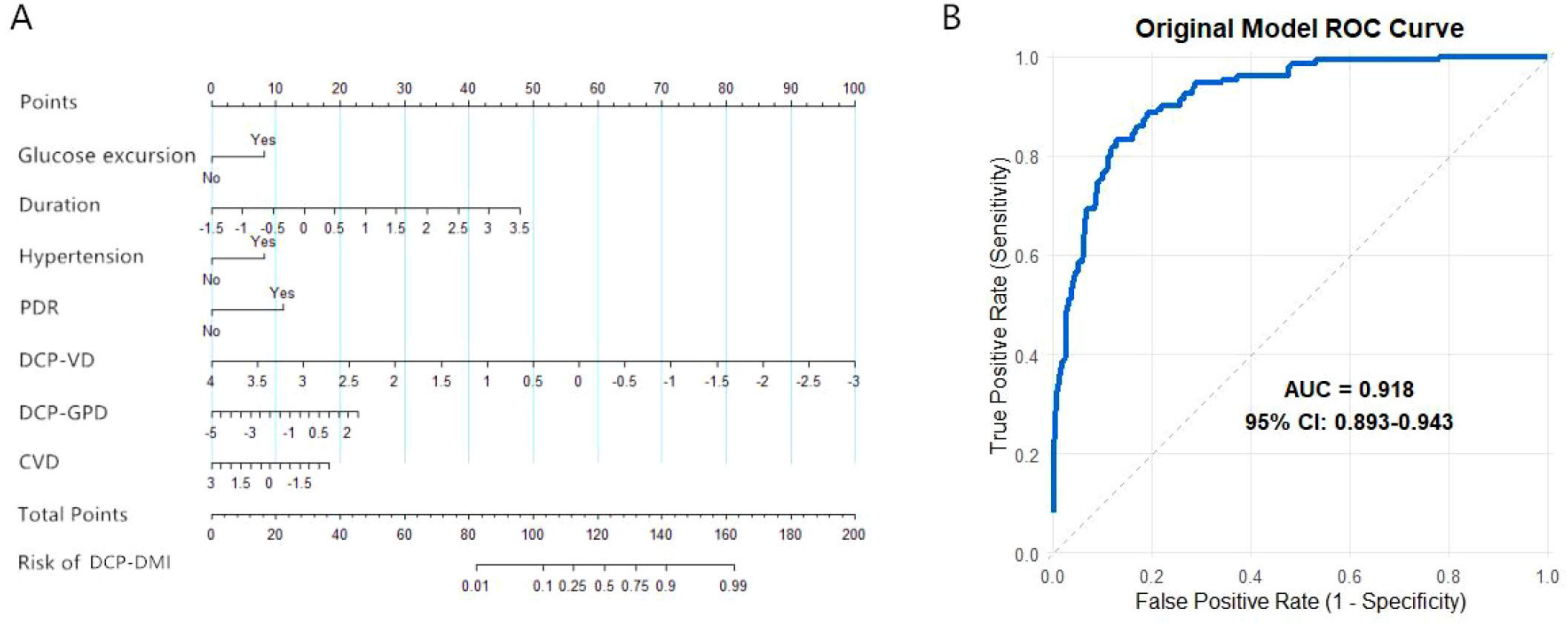
Construction of the prediction model for DCP-DMI occurrence in DR patients. **(A)** Nomogram model for DR patients complicated with DCP-DMI. **(B)** ROC curve of the DCP-DMI prediction model for patients with DR.

Transforming the prediction formula into a nomogram model facilitates clinical application. Patient-specific risk factors correspond vertically to scores on the upper scale, and these scores are summed to obtain a total score. The total score corresponds to the risk probability on the DCP-DMI risk line (**Figure 1A**). The nomogram shows that hypertension, PDR, glucose excursion, duration of diabetes, and DCP-VD are the most influential predictors. These risk factors are non-invasive and easily obtained in outpatient settings, assisting healthcare providers in quickly identifying patients at high risk for DCP-DMI and enhancing chronic disease management. The Hosmer–Lemeshow test assessed the goodness-of-fit of the risk prediction model (χ² = 6.665, P = 0.573), indicating a good fit (P > 0.05). The area under the ROC curve (AUC) was 0.918 (95% CI: 0.893–0.943), demonstrating high discriminative ability (**Figure 1B**).

### 3.4 Internal validation of the nomogram model for DCP-DMI occurrence in DR patients

**Figure 2:**
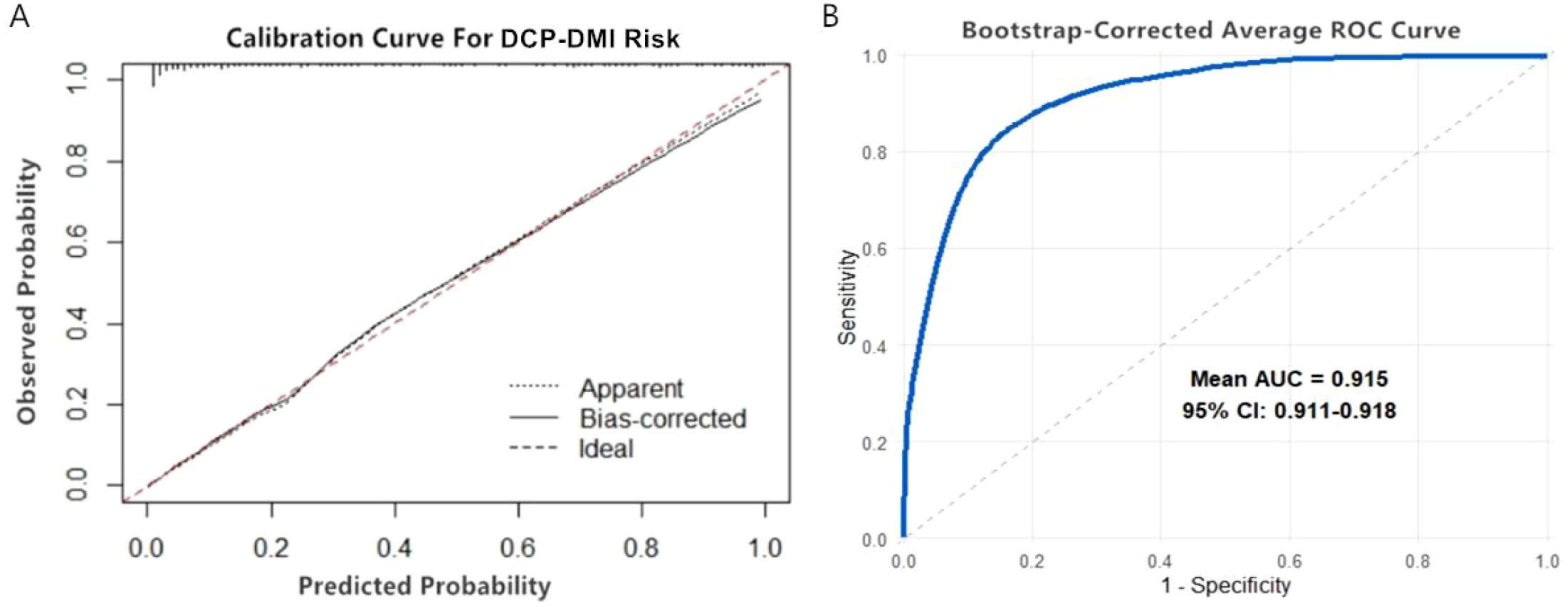
Internal validation of the nomogram model for DCP-DMI occurrence in DR patients. **(A)** Internal validation calibration curve graph. **(B)** Internal validation ROC curve.

The repeatability of the model was validated using the Bootstrap resampling method with 1000 repetitions. After resampling, the Hosmer–Lemeshow test showed χ² = 12.687, P = 0.262, indicating good model fit (P > 0.05). The calibration curve indicated high consistency between predicted and actual probabilities of DCP-DMI occurrence in DR patients (**Figure 2A**). The corrected AUC was 0.915 (95% CI: 0.911–0.918), confirming the model’s strong discriminative performance (**Figure 2B**).

### 3.5 External validation of the nomogram model for DCP-DMI occurrence in DR patients

**Figure 3:**
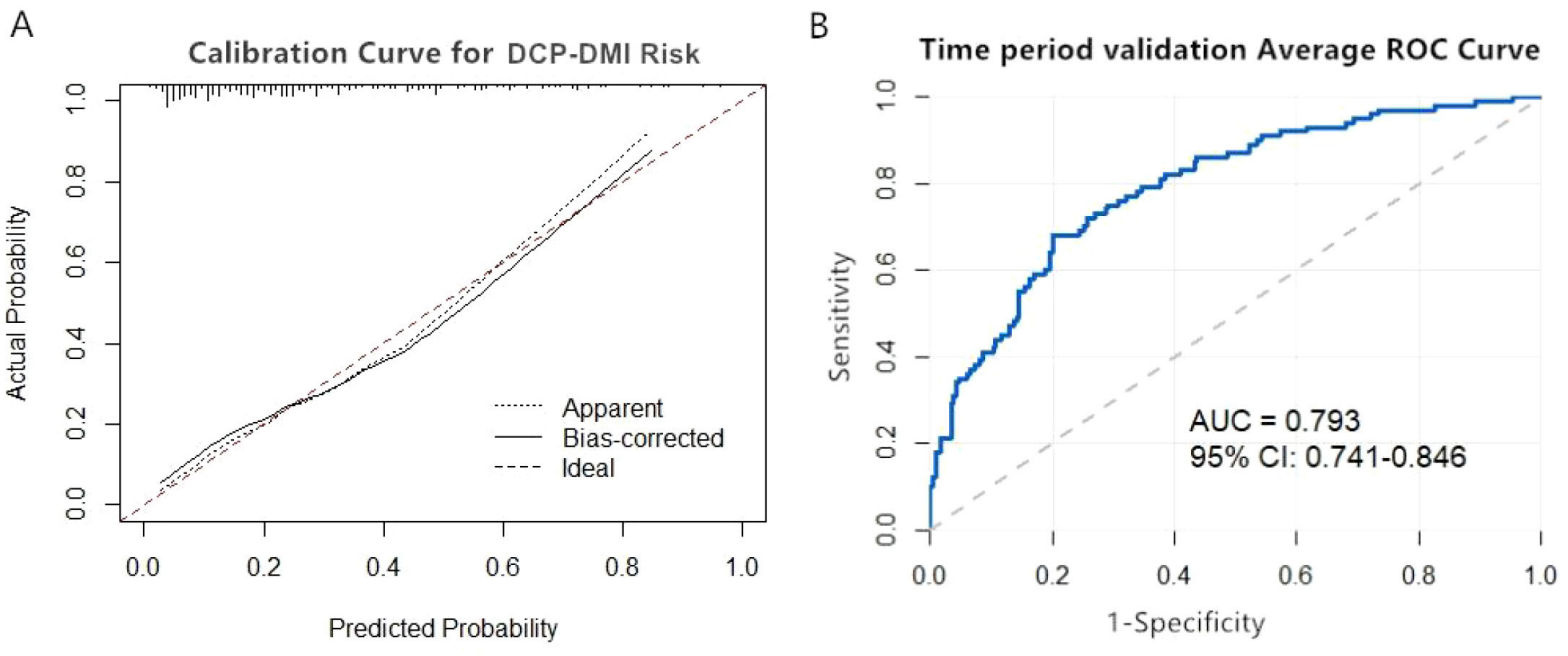
External validation of the nomogram model for DCP-DMI occurrence in DR patients. **(A)** External validation calibration curve graph. **(B)** External validation ROC curve.

The external validity of the model was verified using the time-period validation method. The Hosmer-Lemeshow test showed results of χ² = 10.287, P = 0.246 (P > 0.05), indicating a good model fit. The calibration curve demonstrated that the actual and corrected calibration curves were highly consistent with the calibration reference line. This indicates good agreement between predicted probabilities and observed values of DCP-DMI occurrence in DR patients (**Figure 3A**). The corrected area under the ROC curve (AUC) was 0.793 (95% CI: 0.741–0.846), demonstrating good discriminative capability of the model (**Figure 3B**).

### 3.6 Evaluating the clinical applicability of the nomogram model for DCP-DMI occurrence in DR patients

**Figure 4:**
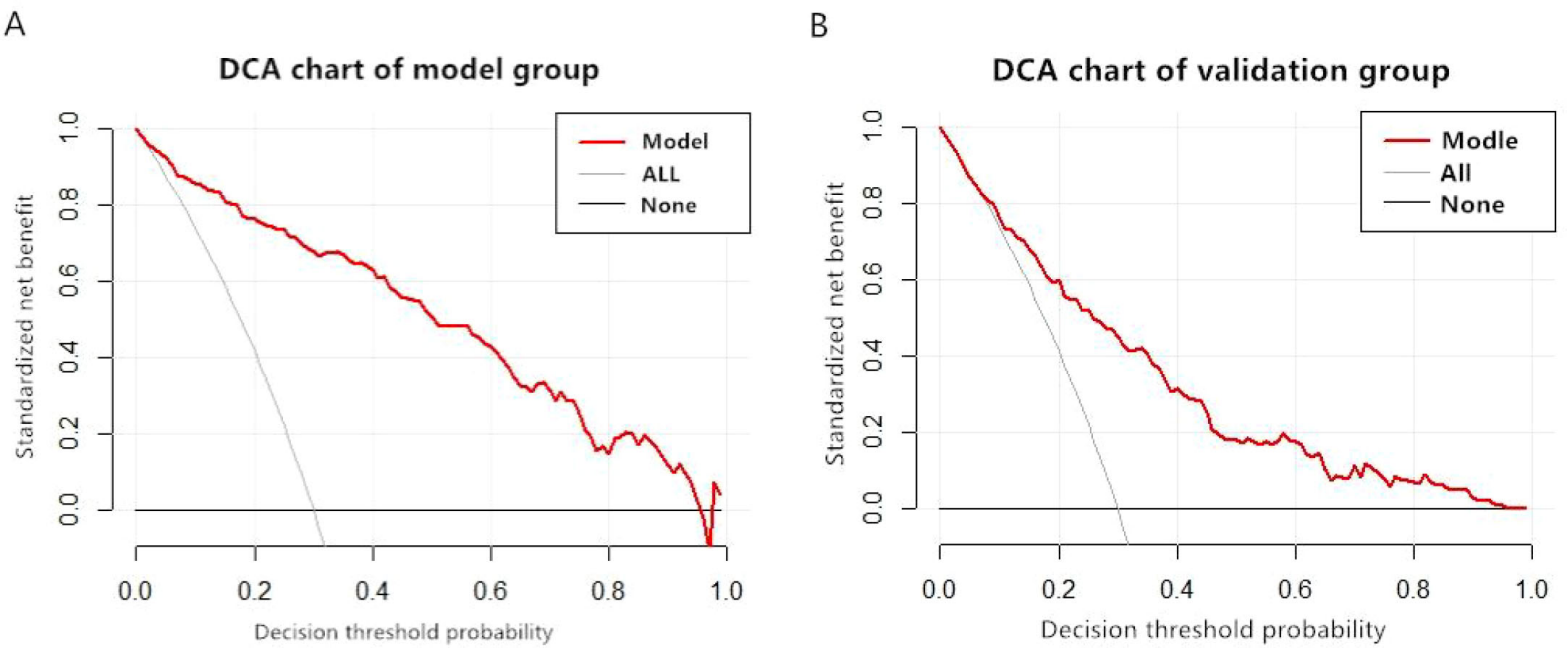
DCA charts of the model group and the validation group. **(A)** DCA charts of the model group. **(B)** DCA charts of the validation group.

DCA was used to evaluate the practical value of the model in both the modelling and validation groups. The DCA plots for the modelling group (**Figure 4A**) and validation group (**Figure 4B**) showed the red curve representing the nomogram consistently above the two reference lines. This indicates a certain net benefit across various thresholds, demonstrating that the constructed model possesses clinical applicability and benefit. However, the predictive performance in the validation group was slightly lower than that in the modelling group.

## 4 Discussion

The initial selection of independent variables for the clinical prediction model was based on literature and clinical experience. To avoid redundancy among OCTA parameters, feature-correlated variables were excluded. These variables included vascular length density, blood flow perfusion density, and vascular perimeter index, which correlate with VD, as well as non-perfusion area percentage and non-perfusion index, which correlate with GPD. According to the literature, the incidence of DMI in diabetic patients is approximately 6%-10% (17). Other studies report a higher incidence of 46%–77% among DR patients, especially those with severe DR (3). This study adopted 30% as the incidence of DCP-DMI for sample size calculation.

Following model construction and validation, this study identified glucose excursion, duration of diabetes, hypertension, PDR, DCP-VD, DCP-GPD, and CVD as independent predictive factors for DCP-DMI risk in DR patients. Comparing ROC and calibration curves from internal and external validation revealed that internal validation performed slightly better. This may be attributed to internal validation using the same dataset as the modelling group, validating reproducibility. Conversely, external validation employed independently collected data, assessing the generalizability of the model. The nomogram constructed in this study translates a multifactorial risk prediction model into an intuitive graphical tool. It allows clinicians to rapidly and individually assess DCP-DMI risk in diabetic patients. By inputting patient-specific clinical indicators, corresponding scores can be summed on the nomogram, directly yielding the probability of developing DCP-DMI. This tool aids clinicians in decisions regarding intensive treatment initiation, scheduling of fundus examinations, and OCTA assessments. The DCA performed in this study indicated that the nomogram curve was consistently above the two reference lines, demonstrating clinical benefit across various thresholds. The constructed nomogram effectively identifies DR patients at high risk for DCP-DMI, facilitating clinical prevention and management strategies. In clinical practice, it is recommended that follow-up intervals occur every 6 to 12 months when the threshold probability is below 20%. For threshold probabilities between 20% and 40%, intervals should shorten to every 3 to 6 months. Threshold probabilities above 40% necessitate comprehensive systemic management and closer follow-up. This nomogram model provides valuable guidance for reducing DCP-DMI occurrence among DR patients and merits clinical application.

Hypertension can increase reactive oxygen species (ROS) and inflammatory factors, causing damage to retinal capillary endothelial cells and pericytes. This results in capillary occlusion and the formation of non-perfused areas (18, 19). Moreover, damage to the DCP is greater than to the SCP (20). Blood glucose fluctuations similarly harm retinal vessels and promote vascular occlusion. Compared with sustained hyperglycaemia, blood glucose fluctuations pose a greater threat to microvessels (21). As the DCP is more susceptible to ischaemia than the SCP, hypertension and blood glucose fluctuations have stronger associations with DCP-DMI than with SCP-DMI. Diabetes duration closely relates to the severity of DR. A duration ≥10 years is a core predictor of PDR. Patients with a diabetes duration of 15 years have a 3.2-fold greater risk of developing PDR compared to those with a 10-year duration (22). Studies indicate that for every additional 5 years, non-perfused regions in the DCP expand by 40%, significantly more than the 25% increase observed in the SCP (23). VD is a core indicator quantifying retinal microcirculation. Compared with systemic or other ocular indicators, VD directly reflects retinal and choroidal blood flow perfusion (24). Reduced CVD is associated with an increased likelihood of DMI (25), since the FAZ completely relies on blood supplied by choroidal capillary networks (26). GPD, an OCTA parameter, quantifies low perfusion or non-perfused areas in the retinal vascular network. In DR patients, GPD assesses the severity of retinal ischaemia (27).

Previous studies have identified diabetes duration, hypertension, age, DME, and PDR as risk factors for DR combined with DMI (28, 29). Our results suggest that age and DME are not predictive factors for DCP-DMI. This discrepancy may arise from two factors: firstly, previous research primarily focused on SCP-DMI, whereas our study targets DCP-DMI. Given differences in blood supply between DCP and SCP, DCP-DMI may be a cause rather than a result of DME. Secondly, OCTA imaging can be influenced by artefacts and image quality when assessing DMI. Such differences in OCTA data may lead to varying results across studies. Although our findings differ from previous studies, our research confirms for the first time that diabetes duration, hypertension, and PDR are risk factors not only for SCP-DMI but also for DCP-DMI. Some studies reported anaemia and hyperlipidaemia as additional risk factors for MI in DR patients (2). Our study did not include these two factors due to their requirement for multiple haematological tests, making them impractical for outpatient-based risk prediction. Nevertheless, our study provides data directly assessing retinal blood flow through OCTA parameters, facilitating more accurate risk assessment.

Given the greater sensitivity of DCP to ischaemia compared with SCP, and its more substantial threat to vision (30), predictive values of risk factors for DCP-DMI exceed those for SCP-DMI. Research on DCP-DMI remains scarce; thus, our results offer valuable clinical references. However, this study has limitations. Firstly, including more reference indicators could improve predictive accuracy. Secondly, the retrospective, single-centre design and reliance on OCTA equipment limit generalizability.

Currently, DMI research remains in its early stages. The pathogenic mechanisms, prevalence, definitions, preventive strategies, and treatments for DMI remain unclear (31). This study provides clinicians with the first predictive model for early identification of DR patients at high risk for concurrent DCP-DMI, enabling timely and effective interventions. Future research will incorporate additional indicators and conduct stratified analyses based on risk probabilities, aiming to establish a more efficient and accurate risk prediction model.

## Data Availability

The raw data supporting the conclusions of this article will be made available by the authors, without undue reservation.
